# The application of “upper-body yoga” in elderly patients with acute hip fracture: a prospective, randomized, and single-blind study

**DOI:** 10.1186/s13018-019-1295-6

**Published:** 2019-08-06

**Authors:** Jinli-Guo, Chaona-Gao, Haifeng-xin, Jiahui-Li, Bing Li, Zhuan Wei, Yiting-Yue

**Affiliations:** 1 Department of Orthopedic, the Second Hospital of Shanxi Medical University, No. 382 of Wuyi Road, Xinghualing District, Taiyuan, 030001 China; 2 Department of Operating Room, the Second Hospital of Shanxi Medical University, Taiyuan, 030001 China; 3 Nursing College of Shanxi Medical University, Taiyuan, 030001 China

**Keywords:** Yoga, Abdominal breathing training, Hip fracture, Forced vital capacity, Peak cough flow, Barthel Index

## Abstract

**Purposes:**

Hip fracture leads to decreased activity and an increased risk of pulmonary complications. The main purpose of this study was to observe the lung capacity, cough capacity of the elderly patient with acute hip fracture, and assess the effects and the feasibility of using a special-designed “upper-body yoga” training to treat elderly patients with hip fracture.

**Methods:**

This was a prospective, randomized, and single-blind study. Eighty-four subjects aged over 65 years were randomly divided into either a control group or a yoga group to undergo an abdominal breathing program or an “upper-body yoga” program until 4 weeks after surgery. The primary outcomes were forced vital capacity/predicted value (FVC%), peak cough flow (PCF), Barthel Index (BI), and the incidence of pneumonia. The secondary outcomes were the rates of right skills and inclination.

**Results:**

Thirty-nine subjects in the yoga group and 40 subjects in the control group completed this study. At the end of the first training week, FVC% (74.14% ± 13.11% vs. 70.87% ± 10.46%, *P* = 0.231) showed no significant difference between the two groups, while the value of PCF (204.80 ± 33.45 L/min vs. 189.06 ± 34.80 L/min, *P* = 0.048) and BI (38.59 ± 8.66 vs. 33.00 ± 9.32, P = 0.009) in the yoga group was higher. After 4 weeks of treatment, FVC%, PCF, and BI were higher in the yoga group (78.83% ± 13.31 % vs. 72.20% ± 10.53%, *P* = 0.016; 216.16 ± 39.29 L/min vs. 194.95 ± 31.14 L/min, *P* = 0.008; 70.77 ± 10.23 vs. 65.75 ± 11.30, *P* = 0.019). One in the control group and nobody in the yoga group was diagnosed with pneumonia. There was no significant difference between the two groups in terms of the rates of right skills, whereas more elderly people preferred the training program of the “upper-body yoga.”

**Conclusion:**

Elderly patients with acute hip fractures are at risk of impaired lung capacity and inadequate cough. “Upper-body yoga” training may improve the quality of daily life, vital capacity, and cough flow in elderly patients, making it a better choice for bedridden patients with hip fracture.

## Introduction

Among elderly patients with hip fracture, pneumonia is known to be a devastating complication in the peri-operative period and during post-discharge recovery, in which the incidence of pneumonia is 4.1–7.0% [1, 2] and its 30-day mortality is 14–27.1% [2, 3]. In the first few days following the onset of fracture, a patient must stay in bed or even take a supine position to avoid aggravating the injury. However, up to 1 month after surgical procedures, the physical activity of the patients remains seriously affected [4], and hence most of the time the patients are restricted to bed rest. During this period, elderly patients tend to breathe with a low tidal volume. Therefore, the absence of regular deep breaths, the presence of postural restriction, and the weakness of expiratory muscles impair the ability of these patients to cough, eventually leading to the loss of ventilation, retention of airway secretion, thus aggravating pre-existing medical conditions (such as chronic obstructive pulmonary disease, diabetes, obesity) and predisposing the elderly patients to atelectasis, pneumonia, and potentially life-threatening respiratory failure. The current literature suggests to reduce the risk of pulmonary complications via early surgery and early mobilization [5–7]. So far, no specific measures have been recommended to prevent and reduce the occurrence of pneumonia in bedridden senile patients. In the routine peri-operative functional exercise, the lower limb movement is emphasized, and the upper limb movement is often ignored.

As a popular form of exercise, yoga is characterized by a combination of slow stretching exercises and breathing techniques, which are suitable for frail elderly patients. Yoga has been proven to play positive roles in the management of mental stress, pain, and chronic diseases [8]. However, the majority of studies on yoga have focused on full-body yoga movements, while few studies have been carried out for bedridden patients. We designed a bed-in yoga program called “upper-body yoga” for elderly bedridden patients. It is hypothesized in this study that, in the early stages of a hip fracture, the special-designed “upper-body yoga” training may help improving lung functions and mobility in elderly patients. The main purpose of this study was to observe the lung capacity and cough capacity of the elderly patients with hip fracture and to assess the clinical effects and the feasibility of using “upper-body yoga” training to treat elderly patients with acute hip fracture.

## Materials and methods

### Design overview

This was a prospective, randomized, and single-blind clinical study, which was approved by the Ethics Committee for Human Research at the Shanxi Medical University.

### Subjects

This study enrolled a total of 84 clinically stable patients with hip fracture who were aged above 65 years. All patients were hospitalized to a teaching hospital within 48 h of hip fracture and stayed in the hospital for more than a week between March 2018 and September 2018. The exclusion criteria were as follows: (1) dependent before fracture; (2) unable to follow medical instructions; (3) also suffering from other injuries; (4) having or may have pulmonary complications before admission; and (5) with gastroesophageal reflux disease, chronic pulmonary disease, heart function insufficiency, acute myocardial infarction, or any other special conditions who are unable to undergo exercise or lung function tests. All patients were randomly divided into either a yoga group (YG) (*n* = 42) or a control group (CG) (*n* = 42). In addition, patients experienced unexpected situations or critical conditions during the treatment were withdrawn from the study.

### Procedures

Forced vital capacity/predicted value (FVC%) and peak cough flow (PCF) were measured with a portable spirometer (CONTEC^TM^) to assess the ventilation functions in the lungs and the ability of the patients to cough. All measurements were performed by the same therapist who was blind, and all patients (blind) were tested in a 30° supine position. In addition, all values were tested three times at different time points to get the maximum value. In addition, the well-known and widely used Barthel Index (BI) was assessed by two nurses who were not strictly blind to evaluate the activity capacity of elderly patients.

On the day of admission (T1), the baseline values of FVC%, PCF, and BI were obtained from all subjects. In the CG, a nurse (not blind) and an audio instructed the patients to carry out routinely abdominal deep breathing training until they could carry out the training independently. In the YG, patients were instructed by a physiotherapist (not blind) and an audio to carry out “upper-body yoga” training until they could carry out the training following the instructional audio. After 7 days of training (T2), the therapist measured the FVC%, PCF, and BI values of all patients and examine if the patients had mastered their respective training. Targeted guidance was implemented to patients who cannot grasp the training methods so as to ensure that all patients could still carry out long-term exercises after discharge. Patients in both groups performed the same exercise regimen of lower body and systemic movement.

Four weeks after surgery, the patients returned to the hospital to review their values of FVC%, PCF, and BI. Moreover, the patients were surveyed about their attitudes towards their training programs and whether they have experienced pneumonia after discharge.

### Training program of abdominal breathing

The way to practice abdominal deep breathing training was as follows: (1) put one hand on the abdomen and the other hand on the chest; (2) inhale deeply through one’s nostrils to raise his/her belly and maintain his/her chest still; and (3) exhale spontaneously through the nostrils. The training was repeated 20 times per session, with 2 sessions carried out per day.

### Training program of “upper-body yoga”

The “upper-body yoga” program was easy to learn and consisted of three phases: a preparatory phase, a main phase, and a final phase. Each phase is described in details below.

Preparatory phase:

Close one’s eyes and concentrate on breathing to inhale slowly and deeply through one’s nostrils, so as to raise his/her belly until the lung is fully expanded. Then, exhale completely through one’s mouth to with a sound of “a~~” for 10 times. Rotate all joints of upper limbs during a 1-min warm-up period.

Main phase:

Complete the following routines with controlled breathing. (a) Inhale deeply and raise one’s arms slowly to 180° from the front of the body, breathe quietly 3–5 times, and exhale completely with arms facing backward. (b) Inhale deeply and raise one’s arms slowly towards the side until his/her palms are facing each other. Then, lean toward the left or right and breathe quietly 3–5 times before exhaling completely with arms facing backward. (c) Inhale and exhale while simultaneously bending the elbows and rotating the shoulder joints as much as possible. Repeat step (a) 5 times and steps (b, c) 10 times (5 times in each direction).

#### Final phase

Close one’s eyes and breathe in and out quietly with his/her hands placing on the abdomen to relax and meditate for 3 min, followed by two quick and forceful breaths using a sound of “ha~”.

## Results

Thirty-nine subjects in the YG (age, 74.10 ± 6.59 years) and 40 subjects in the CG (age, 75.10 ± 6.96 years) completed this study, with 5 patients dropped out due to early discharge, severe electrolyte disturbance, arrhythmia, or hemorrhoea. The baseline characteristics and major clinical interventions used to treat the subjects are shown in Table 1. The median pre-operation time was 4.10 ± 1.43 days in the YG and 3.95 ± 1.48 days in the CG, and the duration of training was 32.26 ± 1.52 days in the YG and 32.00 ± 1.47 days in the CG.

**Table 1:** Baseline characteristics and major clinical interventions of the study population

	CG	YG	t或*χ*^2^	*P*
	*n* = 40	*n* = 39		
Age (years)	75.10 ± 6.96	74.10 ± 6.59	0.654	0.515
Sex, female (*n*, %)	25 (62.50)	22 (56.41)	0.304	0.581
Fracture type				
Femoral neck (*n*, %)	18 (45.00)	23 (58.97)	1.545	0.214
Trochanteric (*n*, %)	22 (55.00)	16 (41.03)		
History of smoking (*n*, %)	8 (20.00)	6 (15.38)	0.288	0.591
ASA (scores)	1.85 ± 0.77	1.92 ± 0.74	0.430	0.668
Waiting time prior to surgery (day)	3.95 ± 1.48	4.10 ± 1.43	0.465	0.643
Duration of training (day)	32.00 ± 1.46	32.26 ± 1.52	0.764	0.447
Surgical procedures				
Hemiarthroplasty (*n*, %)	15 (37.50)	15 (38.46)	0.008	0.930
Hip pinning (*n*, %)	25 (62.50)	24 (61.54)		
Type of anesthesia				
Spinal anesthesia (*n*, %)	35 (87.50)	32 (82.05)	0.455	0.500
General anesthesia (*n*, %)	5 (12.50)	7 (17.95)		
Body mass index (kg/m^2^)	23.22 ± 3.23	23.12 ± 2.93	0.137	0.891

### Spirometer tests

**Fig. 1:**
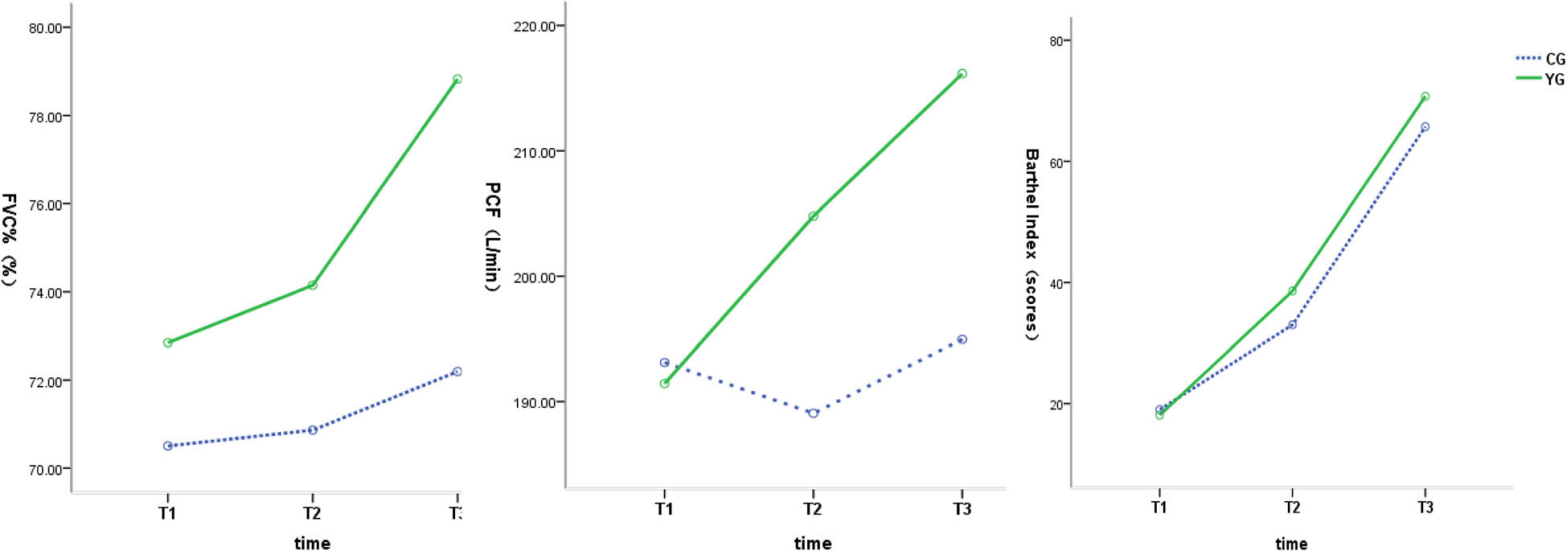
The trend chart of FVC%, PCF, and BI in the two groups at different time. FVC% increased gradually with the extension of time. The difference of PCF at different time points was statistically significant, and the PCF increased gradually with the extension of time. The difference in BI at different time points was statistically significant, and the BI gradually increased with the extension of time

FVC% values at 3 time points are presented in Table 2. The results of repeated measurement ANOVA showed that the difference of FVC% at different time was statistically significant (*F* = 58.152, *P* < 0.001). FVC% increased gradually with the extension of time (Fig. 1). The interaction between time and group was statistically significant (*F* = 18.145, *P* < 0.001). By multiple comparisons of FVC% between the two groups at each time point, it showed that there was no statistically significant difference in FVC% between the two groups on the first day and 1 week after admission (*t* = 0.862, *P* = 0.393; *t* = 1.204,*P* = 0.231). After 4 weeks of surgery, FVC% in YG was significantly higher than that in CG (*t* = 2.437, *P* = 0.016).

**Table 2:** Comparison of FVC% (%)

Group	*n*	T1	T2	T3
YG	39	72.85 ± 14.03	74.14 ± 13.11	78.83 ± 13.31
CG	40	70.51 ± 10.94	70.87 ± 10.46	72.20 ± 10.53

The values of PCF are shown in Table 3. By two-factor repeated measurement ANOVA, the difference of PCF at different time points was statistically significant (*F* = 21.770, *P* < 0.001), and the PCF increased gradually with the extension of time (Fig. 1). The interaction between time and group was statistically significant (*F* = 19.184, *P* < 0.001). Multiple comparisons of PCF between the two groups at each time point showed that on the first day of admission, there was no significant difference in PCF between the two groups (*t* = 0.211, *P* = 0.831). One week after admission and 4 weeks after surgery, PCF in YG was significantly higher than that in CG (*t* = 1.995, *P* = 0.048; *t* = 2.681, *P* = 0.008).

**Table 3:** Comparison of PCF (L/min)

Groups	*n*	T1	T2	T3
YG	39	191.44 ± 33.96	204.80 ± 33.45	216.16 ± 39.29
CG	40	193.13 ± 37.49	189.06 ± 34.80	194.95 ± 31.14

### Barthel Index

The BI of the patients is listed in Table 4. The results of repeated measurement ANOVA showed that the difference in BI at different time points was statistically significant (*F* = 642.977, *P* < 0.001), and the BI gradually increased with the extension of time (Fig. 1). The interaction between time and group was statistically significant (*F* = 5.313, *P* = 0.007). Multiple comparisons of BI between the two groups at each time point showed that on the first day of admission, there was no statistical difference in BI between the two groups (*t* = 0.431, *P* = 0.665). BI in YG was significantly higher than that in CG (*t* = 2.632, *P* = 0.009; *t* = 2.362, *P* = 0.019) at 1 week after admission and 4 weeks after surgery.

**Table 4:** Comparison of Barthel Index (scores)

Group	*n*	T1	T2	T3
YG	39	18.08 ± 6.85	38.59 ± 8.66	70.77 ± 10.23
CG	40	19.00 ± 9.62	33.00 ± 9.32	65.75 ± 11.30

### Adverse events, pneumonia, training skills, and inclination

No adverse event occurred in both groups. Four weeks after surgery, one in CG and nobody in YG was diagnosed with pneumonia. After a week of training, 29 (74.36%) in YG and 33 (82.50%) in CG got the right skills. There was no statistical difference between the two groups (*P* = 0.379). A follow-up at 4 weeks after surgery showed that 33 (84.62%) in YG and 16 (40.00%) in CG admitted that they would like to continue the training protocol in a long period of time. There was a statistically significant difference between the two groups (*P* < 0.001) (Table 5).

**Table 5:** Safety, compliance, and pneumonia (*n*, %)

Group	*n*	Adverse events *n* (%)	Right skill *n* (%)	Compliance *n* (%)	Pneumonia *n* (%)
CG	40	0 (0)	33 (82.50)	16 ( 40.00%)	1 (3.40)
YG	39	0 (0)	29 (74.36)	33 (84.62 %)	0 (0)
*χ* ^2^			0.775	16.688	0.000
P			0.379	< 0.001	1.000

## Discussion

This study showed that the elderly patients who experienced more than 4 weeks of low-intensity “upper-body yoga” training may show higher FVC, PCF, and daily living activity than those who experienced the same period of abdominal breathing training. And such yoga exercise is safe and feasible in the acute phase of hip fracture and subsequent rehabilitation.

Age-related sarcopenia (or decreased muscle mass) is a noticeable problem for elderly patients. Aging reduces respiratory functions as manifested by the decline of pulmonary volume and respiratory muscle tone [9]. Upon hip fracture, the vital capacity and forced expiratory flow in elderly patients confined in a supine position are further reduced. In this study, on the first day of admission elderly patients with healthy lungs showed significantly lower FVC (2.14 ± 0.54 L) and FVC% (71.66% ± 12.53%) than the normal range specified in the guideline of the American Thoracic Society and the values of Cebria et al.’s [10] study on sedentary frail older women (77.7~83.0%). It has been well proven that lower position reduces spirometric values [11, 12]. The low FVC of patients with healthy lungs and hip fracture in the present study may be attributed to postural limitations, physical pain, and stress weakness. As it went to 1 month later, FVC% in both CG and YG increased gradually with the recovery of the disease and the progress of respiratory training. However, the FVC% in YG showed an even bigger increase.

The effective role of yoga in improving pulmonary functions of healthy elderly people and COPD patients has been well recognized [13–15]. However, the effects of different intensity and duration of yoga training on vital capacity and cough capacity are unclear. At present, yoga practices in immobilized elderly have not been well studied.

It is known that the training on abdominal breathing (belly breathing) is the most traditional breathing training method. When belly breathing is repeatedly practiced, the muscle of the diaphragm contracts forcefully to generate a higher tidal volume, thus helping to inflate the lungs and make the diaphragm stronger. Celli et al. [16], Baarends et al. [17], and Petta et al. [18] studied the physiological responses upon unsupported low-intensity static and dynamic exercises of upper limbs (limb elevation, flexion, and extension). These studies revealed that unsupported low-intensity exercises of upper limbs significantly increased ventilation and promotes respiratory muscle function. During arm exercise, accessory muscles of respiration are primarily recruited to positions, which forces the major inspiratory muscle (diaphragm) to increase its contribution to ventilation. Recently, unsupported arm exercises have been widely used in the immediate post-operative period of thoraco-abdominal surgery and in chronic stages of pulmonary diseases [19]. The “upper-body yoga” exercise used in this study is a combination of upper body movement, respiration training (belly inhalation, slowly and fast forced exhalation), and relaxation. It is hypothesized that this training program may be more effective and suitable than traditional belly breathing program in peri-operative management of hip fracture. However, in prior studies, the duration of yoga in majority of the studies was designed over 2 months, these yoga sessions were performed 2~4 times per week [20, 21]. Four weeks seems too short of a time to observe a substantial improvement in respiratory muscles. In this study, the number of yoga sessions was more (2 times/day, 7 days/week). At present, several studies have confirmed the effects of short-term yoga on lung function. In some studies, 4 weeks of yoga have been shown to improve lung function in asthmatic patients [22, 23]. In another study, the elderly patients with COPD showed significantly higher vital capacity and better quality of life after 6 weeks of yoga (3 days/week) [24]. Similarly, we observed that at the end of 4 weeks after surgery, the subjects in YG had higher FVC% than those in CG, indicating the presence of higher ventilation and stronger respiratory muscles in YG. Current research shows that expiratory muscle may be easier to get enhanced by short training sessions than inspiratory muscle. The advantage in YG may be due to a combination of subtle changes in inspiratory muscle and expiratory muscle strength. Unfortunately, we did not have an accurate estimate of respiratory muscle strength. The result above indicates that the combination of upper-body movements and respiration training (“upper-body yoga”) may be more beneficial to improving lung capacity than belly breathing alone.

PCF is the maximum airflow generated during a cough and is dependent on inhaling vital capacity and expiratory muscle tone. Bach et al. [25] suggested that in patients with muscle weakness, a minimum threshold for effective airway clearance was 160 L/min. According to Tzeng and Bach’s [26] experience, when the PCF is < 270 L/min, it is likely to fall below 160 L/min when a patient becomes frailer. In the present study, the baseline PCF in the 30° supine position was 193.13 ± 37.49 L/min in CG and 191.44 ± 33.96 L/min in YG, both of which were significantly lower than 270 L/min. Even after more than 4 weeks of training, the PCF of the patients was still less than 270 L/min. At present, there is no reference values of PCF for different age group, and there are few reports on PCF of the elderly. A study [27] on healthy Brazilian population’s PCF (aged 18~40) showed that PCF decreased significantly with age and the average PCF of men aged 18 and 40 was significantly different (499 L/min vs. 316 L/min). So, advanced age and postural restriction is the primary consideration for the low PCF in this study. These results suggested that when elderly patients were forced to stay in bed, they were at a high risk of sputum retention and lung infection, and hence, it was essential to help remove airway secretions.

In the process of belly breathing program, expiration is passive, expiratory muscles are not activated and hence disuse-atrophy in bed. In the “upper-body yoga” training program, a forced expiratory technology (including slowly and fast forced exhalation) was adopted to improve the main expiratory muscle strength (abdominal and chest muscles). Moreover, upper-body exercises may make the majority of chest and back muscles (accessory expiratory muscles) stronger. Many studies revealed that the training on expiratory muscles increases the tone of expiratory muscles and the rate of voluntary expiratory flow. In Kim’s [28] 4-week training of expiratory muscles in sedentary and healthy elderly subjects, the maximum expiratory pressure (MEP) generated by expiratory muscles quickly increased in the first week of training from 77.14 cmH_2_O to above 90 cmH_2_O, and then gradually increased to 110.83 cmH_2_O in the following weeks. In addition, the peak expiratory flow rate of reflexive cough induced by capsaicin increased from 298.8 ± 130.8 L/min to 480.0 ± 183.6 L/min. In another study, an increase in MEP was observed in 2~4 weeks of training on expiratory muscle strength [29]. However, these studies were carried out using an expiratory pressure threshold device. A recent study [30] observed favorable effects of forced expiratory training on vital capacity and peak expiratory flow in frail and elderly subjects. In this study, the subjects practiced slow expiration using abdominal muscles and a resistance device for 20 min/day in 3 months. In the present study, more than 4 weeks of soothing yoga training increased PCF from 191.44 ± 33.96 L/min to 216.16 ± 39.29 L/min, and it was higher than that of the CG. The results are consistent with previous studies. The higher PCF in YG may be related to stronger inspiratory muscles and expiratory muscles. On the other hand, fast forced expiration maneuver in YG has been considered an effective method to induce tracheobronchial clearance [31, 32].

In the acute phase of fracture, a patient’s quality of daily living is seriously affected. In the first year after hip fracture, only 57% of body functions can be restored [33]. The arms play a central role in daily activities, such as eating, dressing, and washing. In the present study, patients in YG showed higher BI at the points of T1 and T2 than those in CG. A possible explanation for the above results is that upper-limb movement in “upper-body yoga” training makes arms more flexible and stronger. On the other hand, active upper-limb movement can encourage bedridden older people to take care of themselves, thus improving the BI. However, this advantage is more pronounced in the early stages of the disease. With the improvement of the patient’s systemic mobility, the advantage was not more obvious with the extension of time.

In the postoperative follow-up, one in CG was diagnosed with pneumonia, and nobody suffered pulmonary complication in YG. On the one hand, the present study included a relatively small number of patients. On the other hand, the subjects included in this study all had healthy lungs, which put them at a low risk of pulmonary infection.

The most common injuries related to yoga are sprain, strain, and falls, which occur most often in strenuous and challenging training [34]. The “upper-body yoga” program used in the present study was designed by three specialists and was low intensity and easy to learn. No discomfort and adverse events were observed. In addition to physiological changes, aging obviously impairs learning and cognitive functions. In YG, the elderly patients got face-to-face and audio coaching, which made the training easier. Hence, although a yoga program seems more complex than the training of belly breathing, a similar number of elderly patients mastered the yoga skills in this study. In the follow-up interview, most patients considered yoga training fun and relaxing. On the contrary, for people not enjoying the training on abdominal deep breathing, they thought the training was boring. An RCT study comparing the effects of short-term inspiratory threshold training with yoga training in institutionalized frail older adults showed that yoga respiratory training appears to be more effective and well-tolerated in frail older adults, and may therefore be a useful alternative to general breathing training, to improve respiratory function in older population, when whole-body exercise is not possible [35].

### Limitation of the study

There are some limitations in this study. First, only the simplest variables were measured in this study, while more accurate indicators for lung function and respiratory muscle strength are needed. Second, the subjects included in the present study have healthy lungs. There, it is essential to further investigate the effect of “upper-body” yoga breathing program in patients with a high risk of pneumonia. In addition, the participants in this study were not strictly blinded, thus increasing the risk of bias.

## Conclusion

Elderly patients with acute hip fractures are at risk of impaired lung capacity, inadequate cough. “Upper-body yoga” training is a safe and feasible method that may be used to improve the quality of daily life, lung capacity, and cough flow in elderly patients, making it a better choice for bedridden patients with hip fracture or even other medical conditions.

## Data Availability

The dataset supporting the conclusions of this article is included within the article.

## Abbreviations

BI: Barthel Index
CG: Control group
COPD: Chronic obstructive pulmonary disease
FVC: Forced vital capacity
MEP: Maximum expiratory pressure
PCF: Peak cough flow
YG: Yoga group

## References

[1] Boho DD, Sershon RA, Saltzman BM, Darrith B, Della Valle CJ (2018). Incidence, risk factors, and clinical implications of pneumonia after surgery for geriatric hip fracture. J Arthroplasty.

[2] Lv H, Yin P, Long A (2016). Clinical characteristics and risk factors of postoperative pneumonia after hip fracture surgery: a prospective cohort study. Osteoporos Int..

[3] Lawrence VA, Hilsenbeck SG, Noveck H, Poses RM, Carson JL (2002). Medical complications and outcomes after hip fracture repair. Arch Intern Med..

[4] Gherardini S, Biricolti C, Benvenuti E, et al. Prognostic implications of predischarge assessment of gait speed after hip fracture surgery. J Geriatr Phys Ther. 2018;25. 10.1519/JPT.0000000000000144 29373332

[5] Simunovic N, Devereaux PJ, Sprague S (2010). Effect of early surgery after hip fracture on mortality and complications: systematic review and meta-analysis. CMAJ..

[6] Pincus D, Ravi B, Wasserstein D (2017). Association between wait time and 30-day mortality in adults undergoing hip fracture surgery. JAMA..

[7] Carretta E, Bochicchio V, Rocco P, Fabbri G, Laus M, Fantini MP (2011). Hip fracture: effectiveness of early surgery to prevent 30-day mortality. Int Orthop..

[8] Taneja DK (2014). Yoga and health. Indian J Community Med..

[9] Janssens JP, Apache JC, Nicosia LP (1999). Physiological changes in respiratory function associated with ageing. Eur Respir J..

[10] Cebria I, Iranzo MD, Arnall DA (2013). Physiotherapy intervention for preventing the respiratory muscle deteriorationin institutionalized older women with functional impairment. Arch Bronconeumol..

[11] Thakar HM (2015). Spirometric values in sitting, standing and supine position. J Lung Pulm Respir Res..

[12] Vilke GM, Chan TC, Neuman T, Clausen JL (2000). Spirometry in normal subjects in sitting, prone, and supine positions. Respir Care..

[13] Bezerra LA, Melo HFD, Garay AP (2014). Do 12-Week Yoga Program Influence Respiratory Function of Elderly Women?. J Hum Kinet.

[14] Li C, Liu Y, Ji Y, Xie L, Hou Z (2018). Efficacy of yoga training in chronic obstructive pulmonary disease patients: a systematic review and meta-analysis. Complement Ther Clin Pract..

[15] Santaella DF, Devesa CRS, Rojo MR (2011). Yoga respiratory training improves respiratory function and cardiac sympathovagal balance in elderly subjects: a randomised controlled trial. BMJ Open..

[16] Ce lli B, Criner G, Rassulo J (1988). Ventilatory muscle recruitment during unsupported arm exercise in normal subjects. J Appl Physiol (1985)..

[17] Baarends EM, Schols AM, Slebos DJ, Mostert R, Janssen PP, Wouters EF (1995). Metabolic and ventilatory response pattern to arm elevation in patients with COPD and healthy age-matched subjects. Eur Respir J..

[18] Petta A, Jenkins S, Allison G (1998). Ventilatory and cardiovascular responses to unsupported low-intensity upper limb exercise in normal subjects. Aust J Physiother..

[19] Ries AL, Bauldoff GS, Carlin BW (2007). Pulmonary rehabilitation: joint ACCP/AACVPR evidence-based clinical practice guidelines. Chest..

[20] Troosters T, Gosselink R, Janssens W, Decramer M (2010). Exercise training and pulmonary rehabilitation: new insights and remaining challenges. Eur Respir Rev..

[21] Illi SK, Held U, Frank I, Spengler CM (2012). Effect of respiratory muscle training on exercise performance in healthy individuals: a systematic review and meta-analysis. Sports Med..

[22] Sathyaprabha TN, Murthy H, Murthy BT (2001). Efficacy of naturopathy and yoga in bronchial asthma--a self controlled matched scientific study. Indian J Physiol Pharmacol..

[23] Sodhi C, Singh S, Dandona PK (2009). A study of the effect of yoga training on pulmonary functions in patients with bronchial asthma. Indian J Physiol Pharmacol..

[24] Fulambarker A (2012). Effect of yoga in chronic obstructive pulmonary disease. Am J Ther..

[25] Bach JR, Ishikawa Y, Kim H (1997). Prevention of pulmonary morbidity for patients with Duchenne muscular dystrophy. Chest..

[26] Tzeng AC, Bach JR (2000). Prevention of pulmonary morbidity for patients with neuromuscular disease. Chest..

[27] Cardoso FE, de Abreu LC, Raimundo RD (2012). Martins SR, Torquato JA. Evaluation of peak cough flow in Brazilian healthy adults. Int Arch Med.

[28] Kim J, Davenport P, Sapienza C (2009). Effect of expiratory muscle strength training on elderly cough function. Arch Gerontol Geriatr..

[29] Suzuki S, Sato M, Okubo T (1995). Expiratory muscle training and sensation of respiratory effort during exercise in normal subjects. Thorax..

[30] Chigira Y, Miyazaki I, Izumi M (2018). Effects of expiratory muscle training on the frail elderly’s respiratory function. J Phys Ther Sci.

[31] Van HM, Festen J, Beurskens C (1988). Conventional physiotherapy and forced expiration manoeuvres have similar effects on tracheobronchial clearance. Eur Respir J.

[32] Hasani A, Pavia D, Agnew JE (1994). Regional mucus transport following unproductive cough and forced expiration technique in patients with airways obstruction. Chest..

[33] Mariconda M, Costa GG, Cerbasi S (2016). Factors predicting mobility and the change in activities of daily living after hip fracture: a 1-year prospective cohort study. J Orthop Trauma..

[34] Cramer H, Ostermann T, Dobos G (2018). Injuries and other adverse events associated with yoga practice: a systematic review of epidemiological studies. J Sci Med Sport..

[35] Cebrià i, Iranzo MÀ, Arnall DA, Igual Camacho C, Tomás JM (2014). Effects of inspiratory muscle training and yoga breathing exercises on respiratory muscle function in institutionalized frail older adults: a randomized controlled trial. J Geriatr Phys Ther..

