# Correction to: The application of “upper-body yoga” in elderly patients with acute hip fracture: a prospective, randomized, and single-blind study

**DOI:** 10.1186/s13018-019-1369-5

**Published:** 2019-11-04

**Authors:** Jinli Guo, Chaona Gao, Haifeng Xin, Jiahui Li, Bing Li, Zhuan Wei, Yiting Yue

**Affiliations:** 1 Department of Orthopedic, the Second Hospital of Shanxi Medical University, No. 382 of Wuyi Road, Xinghualing District, Taiyuan, 030001 China; 2 Department of Operating Room, the Second Hospital of Shanxi Medical University, Taiyuan, 030001 China; 3 Nursing College of Shanxi Medical University, Taiyuan, 030001 China

**Correction to: J Orthop Surg Res**

**https://doi.org/10.1186/s13018-019-1295-6**

In the original publication of this article [1], there are several errors that need to be corrected as below:

1. In the previous authorship list, “-” in the author names of Jinli-Guo, Chaona-Gao, Haifeng-Xin, and Yiting-Yue should be deleted.
2. The sentence “Four weeks after surgery, the patients returned to the hospital....” should be changed to “Four weeks after surgery (T3), the patients returned to the hospital....”.
3. In Table 1, “t或χ2” should be changed to “t or χ2”.
4. In Table 3, “16 (40.00%)” should be changed to “16(40.00)”, and “33 (84.62%)” should be changed to “33(84.62)”. Moreover, “1(3.40)” should be changed to “1(2.5)”.
5. “T1” should be changed to “T3” in the sentence “In the present study, patients in YG showed higher BI at the points of T1 and T2 than those in CG”.

The original article has been corrected for the author names.

## References

[1] Guo J (2019). The application of “upper-body yoga” in elderly patients with acute hip fracture: a prospective, randomized, and single-blind study. J Orthop Surg Res.

